# The impact of provider-patient communication skills on primary healthcare quality and patient satisfaction in rural China: insights from a standardized patient study

**DOI:** 10.1186/s12913-024-11020-0

**Published:** 2024-05-03

**Authors:** Qiufeng Gao, Bin Zhang, Qian Zhou, Cuiyao Lei, Xiaofei Wei, Yaojiang Shi

**Affiliations:** 1 Center for Experimental Economics in Education, Shaanxi Normal University, Xi’an, 710119 China; 2 School of Economics and Finance, Xi’an Jiaotong University, Xi’an, 710049 China

**Keywords:** Communication skill, Primary healthcare, Standardized patient, Rural China

## Abstract

**Objectives:**

In middle-income countries, poor physician-patient communication remains a recognized barrier to enhancing healthcare quality and patient satisfaction. This study investigates the influence of provider-patient communication skills on healthcare quality and patient satisfaction in the rural primary healthcare setting in China.

**Methods:**

Data were collected from 504 interactions across 348 rural primary healthcare facilities spanning 21 counties in three provinces. Using the Standardized Patient method, this study measured physician-patient communication behaviors, healthcare quality, and patient satisfaction. Communication skills were assessed using the SEGUE questionnaire framework. Multivariate linear regression models and multivariate logistic regression models, accounting for fixed effects, were employed to evaluate the impact of physicians’ communication skills on healthcare quality and patient satisfaction.

**Results:**

The findings indicated generally low provider-patient communication skills, with an average total score of 12.2 ± 2.8 (out of 24). Multivariate regression models, which accounted for physicians’ knowledge and other factors, demonstrated positive associations between physicians’ communication skills and healthcare quality, as well as patient satisfaction (*P* < 0.05). Heterogeneity analysis revealed stronger correlations among primary physicians with lower levels of clinical knowledge or more frequent training.

**Conclusion:**

This study emphasizes the importance of prioritizing provider-patient communication skills to enhance healthcare quality and patient satisfaction in rural Chinese primary care settings. It recommends that the Chinese government prioritize the enhancement of provider-patient communication skills to improve healthcare quality and patient satisfaction.

## Introduction

The provision of medical services inherently involves a dynamic exchange between healthcare providers and patients [1]. Effective physician-patient communication holds pivotal importance, influencing treatment adherence, overall health outcomes, and patient satisfaction [2, 3]. Physicians with adept communication skills are better equipped to understand patient symptoms and treatment preferences, thus facilitating more informed diagnoses and treatment plans [2]. Moreover, proficient communication nurtures a sense of support and care, enhancing both patient satisfaction and compliance with medical advice [4]. Empirical evidence consistently underscores the substantial impact of physician-patient communication on the quality of medical services, directly affecting health outcomes and patient satisfaction.

Despite its critical role, deficiencies in provider-patient communication persist, particularly in low- and middle-income countries. Enhancing physician communication skills emerges as a potential avenue to elevate healthcare quality and patient satisfaction in these settings. Physicians in low- and middle-income countries often prioritize treatment over communication, especially in primary health facilities [5, 6]. Studies conducted in Pakistan, Bangladesh, and India reveal a widespread lack of recognition among physicians regarding the importance of communication, with many failing to employ proper communication skills during patient interactions [7–9]. Additionally, some physicians in primary health facilities exhibit negative attitudes and low motivation to improve their communication skills [9]. This oversight results in inadequate consideration of patient needs and expectations, potentially compromising clinical performance and patient satisfaction. Notably, primary physicians in these low- and middle-income countries exhibit lower levels of healthcare quality and patient satisfaction, evidenced by inaccurate diagnoses, the overuse of antibiotics, and diminished patient satisfaction rates [10–14]. Recognizing and addressing these communication gaps is imperative for enhancing healthcare delivery and patient outcomes in these regions.

This issue is particularly pronounced within China, a middle-income country, where challenges in effective communication within its primary healthcare system in rural areas are prevalent. Similar to their counterparts in other low- and middle-income countries, a substantial number of physicians in China face deficiencies in communication skills and frequently neglect patient interaction [15–17]. This challenge is more pronounced among primary healthcare physicians, who grapple with a demanding workload and limited professional rewards [18]. Compounding this issue, primary physicians in rural areas often exhibit lower levels of education and qualifications [19, 20]. Furthermore, research in primary healthcare indicates that only 4.05% of primary care physicians have completed junior college or higher education level, while also holding a practicing physician certificate or practicing assistant physician certificate [21]. This further hinders their ability to leverage knowledge and communication skills effectively [19], resulting in suboptimal healthcare quality in rural China. For example, the results of Sylvia’s study revealed that rural primary physicians achieved only a 26% accuracy rate in diagnoses and prescribed unnecessary and harmful medications in 64% of interactions [20]. Consequently, a pervasive lack of trust in rural primary physicians has emerged among rural residents, leading to significantly lower levels of patient satisfaction compared to their urban counterparts [22, 23].

Our study contributes to the literature in several significant ways. Firstly, we analyze the link between patient-physician communication skills and healthcare quality in the context of primary healthcare centers in rural China. While inadequate physician-patient communication remains a recognized barrier to improving clinical performance and patient satisfaction in China [2, 18, 24], empirical studies linking physician communication skills to these outcomes in primary care settings are notably lacking. Secondly, previous research has predominantly focused on primary care settings in urban areas of China, neglecting these associations in rural settings [1, 25]. Thirdly, we provide new empirical evidence employing the Standardized Patient (SP) method to analyze the impact of communication skills on healthcare quality in rural primary care settings. It is crucial to recognize that determinants of healthcare quality and patient satisfaction typically arise from characteristics inherent to both the patient and the health provider [12, 20]. However, existing investigations heavily rely on patient perception and recall-based surveys, introducing potential bias and inaccuracies in findings and failing to control for patient-related confounding factors [10]. To overcome these limitations, the SP method has gained prominence in developing countries for assessing physicians’ communication behaviors and healthcare quality [18]. This approach involves simulated patients seeking healthcare unannounced in real-world settings, thereby minimizing recall and subjective biases. Furthermore, it aids in controlling biases within patient populations, facilitating valid quality comparisons across diverse healthcare providers [26]. While Su et al.’s study has utilized the SP method to explore associations between physicians’ communication skills and clinical performance in urban areas of China, rural areas have been notably absent. Consequently, in this study, we employ the SP method to investigate the impact of provider-patient communication skills on healthcare quality and patient satisfaction within primary healthcare settings in rural areas of China.

## Method

### Sample study design and participants

This cross-sectional study was conducted in rural areas of China. Local rural residents, trained as Standardized Patients (SPs), were engaged to gather objective information on healthcare quality, patient satisfaction, and communication behaviors between physicians and patients in primary healthcare settings. Previous studies have demonstrated the suitability of the SP method for assessing communication skills, where SP satisfaction is indicative of patient satisfaction [18, 27, 28].

The study surveyed 348 rural primary health facilities, randomly selected from three provinces: Sichuan, Shaanxi, and Anhui, representing Southwestern, Northwestern, and Eastern China, respectively. These rural primary health facilities consist of township health centers and village clinics, serving as the frontline of China’s rural health system. They typically bear the primary responsibility for addressing the majority of prevention, treatment, and management of common diseases within rural communities [20, 29]. Employing a multi-level random sampling method, primary physicians from one designated prefecture in each of the three sample provinces were involved. Within the 24 counties in the three sample prefectures, 21 counties were randomly selected. Subsequently, 209 township health centers and 139 village clinics were randomly chosen for the study sample.

### Procedures

Trained SPs performed three typical rural cases (diarrhea, tuberculosis, and unstable angina) in a standardized manner. After training and selection, 61 SPs were randomly assigned to seek healthcare unannounced in sampled primary health facilities in August 2015. The distribution included three SPs successively allocated to one township health center, and one SP to one village clinic. Due to primary physicians’ absence during visits, 504 disease cases were completed involving 413 primary physicians.

Alongside the SP survey, additional surveys collected information on physicians, facilities, and visits. Physicians also participated in Vignettes tests as part of the comprehensive evaluation of their clinical knowledge. Vignettes, regarded as a valid quality assessment tool for clinical knowledge in healthcare in developing countries [30], were administered to the sample physicians using the same cases as those presented to the Standardized Patients. This dual assessment approach allowed for the evaluation of physicians’ clinical performance via the SP survey and their levels of clinical knowledge through the Vignettes test.

### Physician-patient communication

The communication skills of primary physicians were assessed using the SEGUE questionnaire, a widely-used tool encompassing five dimensions: set the stage, elicit information, give information, understand the patient’s perspective, and end the encounter [31]. We utilized a Chinese version of SEGUE, previously validated for medical students and primary physicians [17, 32]. Entry 20 of the SEGUE questionnaire (identifying the efforts, accomplishments, or difficulties the patient had to overcome) was removed from the analysis because this study did not require standardized patients to express their efforts, accomplishments, and difficulties to their physicians. The recorded communication behaviors between primary physicians and SP patients were systematically scored using the SEGUE framework (more details about the data analysis can be found in [17]). Each SEGUE item had binary responses, “Yes” or “No”, corresponding to scores of 1 and 0, respectively. The overall score for each encounter across different communication dimensions was computed by summing the scores for each dimension, with higher scores indicating stronger provider-patient communication skills. The SEGUE questionnaire’s five dimensions demonstrated high internal consistency, as indicated by a Cronbach’s α value of 0.63, affirming its reliability and effectiveness.

### Healthcare quality and patient satisfaction

Our study primarily focused on healthcare service quality, which comprises two crucial dimensions: healthcare quality delivered by providers and patient satisfaction. One dimension evaluated healthcare service quality delivered by providers based on criteria such as the percentage of completed recommended questions and exams, correct diagnosis, and correct treatment, commonly utilized in healthcare quality research [20, 29]. We assessed healthcare quality based on these dimensions in the interactions between primary physicians and SPs. According to predetermined standards, a correct diagnosis or appropriate treatment by the physician was recorded as “Yes” and assigned a value of 1; otherwise, it was assigned a value of 0. Simultaneously, a thorough assessment of these three dimensions of physicians’ clinical knowledge was conducted.

The other dimension is patient feedback—patient satisfaction, which is exceedingly important. Previous studies have indicated that patient satisfaction can influence patients’ behavioral intentions and lead to better health outcomes, making it a vital indicator of the quality of care [33, 34]. To measure patient satisfaction, SPs were asked to evaluate their satisfaction with the visited physician after each consultation. Specifically, SPs, acting as patients, were inquired whether they would choose to revisit the same physician when feeling unwell. Patient satisfaction was standardized by recording the patient’s response to the question, “If you were to see a doctor next time, would you want to see this doctor again?”. A response of 1 denoted “Yes,” while 0 indicated “No.”

### Covariates

We also developed additional measures to capture characteristics of physicians and facilities, as well as characteristics of visits. These covariates include gender (1 = male, 0 = female), age (years), education level (1 = college or higher, including adult education; 0 = below college), physician certification (1 = practicing physician certificate or practicing assistant physician certificate; 0 = village physician qualification certificate or no formal qualification including nurse qualification or pharmacist qualification), semi-annual appraisals (1 = yes, 0 = no), number of medical training sessions attended last year (times), the value of medical instruments (RMB 100000), and the waiting (minutes) and consultation time (minutes) during SP visits. Additionally, the physicians’ clinical knowledge level is assessed through the Vignettes test across three dimensions, including the percentage of completed recommended questions and exams, knowledge of correct diagnosis (1 = yes, 0 = no), and correct treatment (1 = yes, 0 = no).

### Statistical analysis

To examine the impact of communication skills on healthcare quality and patient satisfaction, multivariate linear regression models and multivariate logistic regression models^1^ were respectively employed for continuous outcome variables and classification outcome variables. The regression models are specified as:

1 $${E(Y_{1ij}})={\beta }_{0}+{\beta }_{1}{C}_{ij}{+\beta }_{k}{X}_{ij}+{\beta }_{k+1}{k}_{ij}+{\gamma }_{i}+{\delta }_{m}+{\mu }_{ij}$$

2 $${E(Y_{2ij}})={\rho }_{0}+{\rho }_{1}{C}_{ij}{+\rho }_{k}{X}_{ij}+{\gamma }_{i}+{\delta }_{m}+{\mu }_{ij}$$

where ${ Y}_{1ij}$ indicates the indicators of healthcare quality (including the percentage of completed recommended questions and exams, correct diagnosis, and correct treatment) observed during the interaction of a particular SP with physician $j$ for disease case $i$. Similarly, ${Y}_{2ij}$ indicates patient satisfaction during the same interaction with physician $j$ for disease cases $i$. ${C}_{ij}$ respectively represents the overall score of physicians $j$ of disease cases $i$ regarding communication skills and their performance in five dimensions. ${ X}_{ij}$ comprises variables about physicians, facilities, and visits during the interaction of a particular SP with physician $j$ for disease case $i$. ${\gamma }_{i}$ and ${\delta }_{m}$ indicate the fixed effect of disease cases and SPs. ${\mu }_{ij}$ is the error term. Considering that observations of the same doctor may be correlated, we also adopted the robust clustering standard error with the physician. For estimation (1), to control unobservable heterogeneity, the clinical knowledge level ${k}_{ij}$ is also controlled in the analysis, which includes three dimensions through the Vignettes test: knowledge in percentage of completed recommended questions and exams, knowledge of correct diagnosis, and correct treatment provided by physician $j$ for sample case $i$.

Furthermore, to assess whether the impact of communication skills on healthcare quality and patient satisfaction varies among different subgroups, we introduced interactions for heterogeneity analysis of physicians’ training and level of clinical knowledge. The regression models are specified as:

3 $$\eqalign{ E({Y_{3ij}}) & = {\varphi _0} + {\varphi _1}\;{{\text{C}}_{ij}} + {\varphi _2}\;knowledge \cr & \quad + {\varphi _3}\;{C_{ij}}\,*\,knowledge + {\varphi _k}{X_{ij}} + {\gamma _i} + {\delta _m} + {\mu _{ij}} \cr}$$

4 $$\eqalign{ E\left( {{Y_{4ij}}} \right) & = {\alpha _0} + {\alpha _1}\;{{\text{C}}_{ij}} + {\alpha _2}\;training + {\alpha _3}\;{{\text{C}}_{ij}}\,*\,training \cr & \quad + {\alpha _k}{X_{ij}} + {\gamma _i} + {\delta _m} + {\mu _{ij}} \cr}$$

where ${Y}_{3ij}$ and ${Y}_{4ij}$ both indicate the indicators of healthcare quality and patient satisfaction during the SP’s visit to physician $j$ in sample case $i$, and $?3$ and${ \alpha }_{3}$ indicate heterogeneity of clinical knowledge level and physicians’ training of physicians $j$ of disease cases $i$. The clinical knowledge level is assessed by the knowledge of correct treatment; a correct treatment indicates a higher level and is assigned a value of 1, whereas an incorrect treatment indicates a lower level and is assigned a value of 0. The training is categorized as frequent or infrequent based on the average number of medical training sessions attended by the sample of doctors in the last year. Those who attended more than the average number are defined as frequent and assigned a value of 1, while those who attended less than or equal to the average number are defined as infrequent and assigned a value of 0.

## Results

### Characteristics of sample physicians, facilities, and visits

In general, among the 413 primary physicians included in the study, 69.7% were affiliated with township health centers **(**Table 1, Row 1). The majority of the sampled physicians (87.4%) were male, with an average age of around 45 (Rows 2 & 3). Furthermore, approximately 47.9% and 43.6% of the physicians had completed a college or higher education and possessed a practicing physician certificate or practicing assistant physician certificate, respectively (Rows 4 & 5). A total of 41.2% of the providers underwent semiannual appraisals, and the mean times of medical training was 4.3 (Rows 6 & 7). The average estimated value of medical equipment within the surveyed facilities was approximately RMB 500,000 (Row 8). During the 504 interactions between SPs and primary physicians, the average waiting time was about 5.0 minutes (Row 9). On average, primary physicians spent only 3.2 minutes with each SP patient (Row 10). Moreover, the clinical knowledge assessed by the Vignettes test showed that physicians completed 23.9% of recommended questions and exams, 61.1% arrived at a correct diagnosis, and 64.1% arrived at a correct treatment (Table 1, Rows 11 to 13).

**Table 1:** Sample characteristics of basic information and clinical information

Variable	Mean$\text{ }\pm \text{ }$sd or *n* (%)
**Characteristics of physicians and facilities (** ***n*** ** = 413)**	
1. Provider from township health center	288 (69.7)
2. Gender (male = 1)	361 (87.4)
3. Age (years)	45.4 ± 10.5
4. Education level (college or higher, including adult education = 1)	198 (47.9)
5. Physician certification (possession of physician certificate or practicing assistant physician certificate = 1)	180 (43.6)
6. Semi-annual appraisals (yes = 1)	170 (41.2)
7. Number of medical training sessions attended in the preceding year (times)	4.3 ± 5.7
8. Value of medical instruments (RMB100 000)	5.0 ± 6.6
**Characteristics of visits (** ***n*** ** = 504)**	
9. Waiting time (minutes)	5.0 ± 7.9
10. Consultation time (minutes)	3.2 ± 3.5
**Clinical knowledge level (** ***n*** ** = 504)**	
11. Knowledge in percentage of completed recommended questions and exams (%)	23.9 ± 11.5
12. Knowledge of correct diagnosis (yes = 1)	308 (61.1)
13. Knowledge of correct treatment (yes = 1)	323 (64.1)
**Healthcare quality (** ***n*** ** = 504)**	
14. Percentage of completed recommended questions and exams (%)	21.1 ± 11.4
15. Correct diagnosis (yes = 1)	214 (42.5)
16. Correct treatments (yes = 1)	200 (39.7)
**Patient satisfaction (** ***n*** ** = 504)**	
17. Patient revisit preference (yes = 1)	299 (59.3)
**Communication skills (** ***n*** ** = 504)**	
18. Communication skills total score (0 ∼ 24 scores)	12.2 ± 2.8
**Five dimensions (** ***n*** ** = 504)**	
19. Set the stage (0 ∼ 5 scores)	2.7 ± 0.7
20. Elicit information (0 ∼ 10 scores)	5.6 ± 1.5
21. Give information (0 ∼ 4 scores)	2.0 ± 0.9
22. Understand the patient’s perspective (0 ∼ 3 scores)	1.3 ± 0.7
23. End the encounter (0 ∼ 2 scores)	0.5 ± 0.5

### Healthcare quality and patient satisfaction of rural primary physicians

During the SP consultations, primary physicians only addressed approximately one-fifth of the recommended questions and exams, averaging 21.1% (Table 1, Row 14). In general, diagnoses were entirely incorrect in 57.5% of interactions (Row 15). Across the three disease cases, treatments were correct in 39.7% of interactions (Row 16). In addition, in terms of patient satisfaction, 40.7% of SPs expressed dissatisfaction with the physicians they visited and indicated that they would not choose to see the physician again (Row 17).

### Communication skills of rural primary physicians

In general, the comprehensive assessment of communication skills among primary healthcare providers in rural China yielded an overall score of 12.2 out of 24 (Table 1, Row 18). This signifies that, on average, primary physicians achieved only about 50% completion of all SEGUE communication tasks. Delving into the five dimensions of assessment, primary physicians scored 54% (2.7 out of 5) in setting the stage, 56% in eliciting information, and 50% in giving information (Rows 19 to 21). In contrast, primary physicians exhibited comparatively weaker performance in understanding the patient’s perspective and ending the encounter. In these dimensions, the average score was around 1.3 (out of 3) and 0.5 (out of 2), respectively (Rows 22 & 23).

### Impact of physicians’ communication skills on healthcare quality and patient satisfaction

The results of multivariate linear regression and multivariate logistic regression analysis show a significantly positive impact of physicians’ communication skills on healthcare quality and patient satisfaction (Tables 2 and 3). Firstly, after adjusting for physician, facility, and visit characteristics, clinical knowledge of primary physicians, and accounting for the fixed effects of SPs and disease cases, we observed that for each one-unit increase in total communication skills scores, there was a substantial 40.2% improvement (*P* < 0.001) in the percentage of completed recommended questions and exams, and an 18.6% increase (*P* = 0.001) in the likelihood of providing a correct diagnosis. Simultaneously, higher levels of communication skills had a greater impact on patient satisfaction.

**Table 2:** The impact of provider-patient communication skills on healthcare quality (*n* = 504)

	Percentage of completed recommended questions and exams			Correct diagnosis			Correct treatment		
	β value	T value	*P* value	Β value	OR value	*P* value	Β value	OR value	*P* value
Communication skills total score	0.402	10.102	< 0.001	0.170	1.186	0.001	0.101	1.107	0.051
**Four dimensions**									
Set the stage	0.121	3.234	0.001	−0.161	0.852	0.387	−0.091	0.913	0.608
Elicit information	0.262	6.662	< 0.001	0.217	1.242	0.015	0.069	1.072	0.452
Give information	0.099	2.476	0.014	0.300	1.350	0.042	0.343	1.409	0.016
Understand the patient’s perspective	0.087	2.441	0.015	0.169	1.184	0.356	0.081	1.085	0.621

*Notes*: As the “end the encounter” dimension of provider-patient communication skills occurs after physicians make diagnosis and treatment decisions, it does not directly influence clinical quality. Therefore, the regression for clinical quality did not include the score of “end the encounter”. All the above regressions controlled for physician, facility, and visit characteristics, as well as the case and standardized patient fixed effects. The first model controlled the percentage of completed questions and exams through the Vignettes test, the second one controlled the knowledge of correct diagnosis, and the third one controlled the knowledge of correct treatment. Standard errors were clustered at the physician level.

Secondly, when examining different dimensions of communication skills, we found that all these communication skills significantly impacted healthcare quality and patient satisfaction (Tables 2 and 3). Specifically, each dimension of communication skills exhibited a positive impact on clinical performance in terms of the percentage of completed recommended questions and exams (*P* < 0.05). Moreover, improvements in provider-patient communication skills in eliciting information and giving information had an impact on a higher rate of correct diagnosis among rural primary providers(*P* < 0.05). However, among the four dimensions of communication skills, only giving information significantly positively impacted correct treatment (*P* < 0.05). Additionally, the three dimensions of skills — eliciting information, giving information, and ending the encounter — also played significant roles in enhancing patient satisfaction (*P* < 0.05).

**Table 3:** The impact of provider-patient communication skills on patient satisfaction (*n* = 504)

	Patient satisfaction		
	Β value	OR value	*P* value
Communication skills total score	0.205	1.227	< 0.001
**Five dimensions**			
Set the stage	−0.235	0.791	0.164
Elicit information	0.226	1.254	0.009
Give information	0.361	1.435	0.008
Understand the patient’s perspective	0.174	1.190	0.314
End the encounter	0.546	1.727	0.013

*Notes:* The above regressions all controlled for physician, facility, and visit characteristics, as well as the case and standardized patient fixed effects. Standard errors were clustered at the physician level.

### Heterogeneity analysis

The regression analysis further found that the impact of provider-patient communication on healthcare quality and patient satisfaction was influenced by physicians’ clinical knowledge level and training (Table 4). The impact of physicians’ communication skills on healthcare quality was more pronounced for primary physicians with lower levels of clinical knowledge (*P* < 0.01, Table 4, Row 1). When examining the different dimensions of communication skills, we observed that lower levels of physicians’ clinical knowledge enhanced the positive effect of communication skills in giving information (*P* < 0.05, Table 4, Row 4), but higher levels strengthened the positive effect of communication skills in eliciting information (*P* < 0.05, Table 4, Row 3). However, when considering different physicians’ training, the results were less consistent. Although the differences were not significant in overall communication skills scores, we observed a stronger effect of communication skills on physicians who had received more frequent training, particularly in the dimension of eliciting information (*P* < 0.05, Table 4, Row 9), and setting the stage and understanding the patient’s perspective (*P* < 0.01, Table 4, Row 8 & Row 11).

**Table 4:** Heterogeneity analysis by physicians’ clinical knowledge level and training (*n* = 504)

	Percentage of completed recommended questions and exams	Correct diagnosis	Correct treatment	Patient satisfaction
**Table A: By physicians’ clinical knowledge level**				
1. Communication skills total score × knowledge level	−0.009**	0.120	−0.107	0.047
	(0.003)	(0.091)	(0.091)	(0.083)
**Five dimensions**				
2. Set the stage × knowledge level	−0.003	−0.103	−0.409	0.147
	(0.013)	(0.353)	(0.379)	(0.327)
3. Elicit information × knowledge level	−0.003	0.407*	−0.170	−0.070
	(0.006)	(0.178)	(0.188)	(0.180)
4. Give information × knowledge level	−0.024*	0.024	0.006	−0.041
	(0.010)	(0.276)	(0.296)	(0.278)
5. Understand the patient’s perspective × knowledge level	−0.012	−0.418	0.259	0.493
	(0.013)	(0.372)	(0.393)	(0.383)
6. End the encounter × knowledge level				0.325
				(0.453)
**Table B: By physicians’ training**				
7. Communication skills total score × training	0.003	0.030	0.089	0.109
	(0.004)	(0.103)	(0.098)	(0.088)
**Five dimensions**				
8. Set the stage × training	0.005	0.429	−0.008	0.675*
	(0.013)	(0.379)	(0.392)	(0.344)
9. Elicit information × training	0.011*	0.187	0.121	−0.021
	(0.006)	(0.204)	(0.194)	(0.175)
10. Give information × training	−0.002	−0.318	−0.272	−0.277
	(0.010)	(0.303)	(0.282)	(0.263)
11. Understand the patient’s perspective × training	−0.019	−0.286	0.684	0.840*
	(0.013)	(0.405)	(0.398)	(0.363)
12. End the encounter × training				−0.487
				(0.465)

*Notes:* The above regressions all controlled for physician, facility, and visit characteristics, as well as the case and standardized patient fixed effects. Clustering errors at the physician level are in parentheses. ** *p* < 0.01, * *p* < 0.05.

## Discussion

To the best of our knowledge, this study is the first to employ the SP method to investigate the impact of physician communication behaviors on healthcare quality and patient satisfaction within rural primary care settings in China. Drawing insights from 504 disease cases across 348 rural primary health facilities, our findings underscore a general deficiency in doctor-patient communication quality, significantly contributing to lower clinical performance and patient satisfaction. Notably, the impact of physicians’ communication skills on healthcare quality, as well as patient satisfaction, is more pronounced among primary physicians with lower clinical knowledge or those who undergo frequent training.

### Poor communication skills and low levels of healthcare quality and patient satisfaction in rural China

The data extracted from interactions between SPs and physicians in rural primary health facilities in China highlight significant inadequacies in physicians’ communication skills. These shortcomings are particularly notable in light of the prevalent human capital disparities among primary physicians in China and other low- and middle-income countries, notably concerning lower educational attainment and certification levels [11, 12, 19]. This observation not only aligns with findings within China but also resonates with similar challenges observed in other low- and middle-income countries, such as Brazil and India, underscoring a widespread issue of insufficient communication skills among physicians during patient interactions [7–9]. Furthermore, the observed low scores in communication skills among rural primary physicians in this study stand out as notably inferior to those reported in urban primary care settings in China [18], as well as when compared to the communication skills of medical students and physicians globally. Specifically, the communication scores of rural primary physicians were 13.38% lower than China’s community general practitioners, and significantly lagging behind American general practitioners (24.64%) [31, 35]. Given the limited human resources and inadequate communication skills of rural primary physicians, the observed low levels of clinical performance, including a 42.5% rate of correct diagnoses and a 39.7% rate of proper treatments, are not surprising. These findings echo those from previous studies conducted in rural primary care settings in China [20, 27].

### Impact of communication skills on healthcare quality and patient satisfaction

Consistent with prior research, our regression analysis demonstrates the significant impact of communication behaviors exhibited by rural primary physicians on both clinical performance and patient satisfaction [18, 25]. The survey data provides compelling evidence that higher-quality physician-patient communication improves healthcare quality outcomes and heightens patient satisfaction. Established studies emphasize that physicians with superior communication skills can more effectively elicit, process, and understand information, ultimately delivering a higher standard of healthcare service [31, 32]. Additionally, physicians with enhanced communication abilities can help alleviate patient anxiety and negative emotions, ultimately leading to heightened patient satisfaction [36].

In the context of rural Chinese primary physicians, specific dimensions of communication skills—eliciting information, giving information, and ending the encounter—emerge as particularly significant for enhancing healthcare quality and patient satisfaction. Upon exploring the reasons behind this phenomenon, it becomes evident that the processes of eliciting information and giving information play a crucial role in enabling primary physicians to efficiently comprehend and process the information gathered during patient interactions. The exchange of valuable information between physicians and patients contributes to heightened patient satisfaction and increased cooperation with healthcare procedures [37]. Furthermore, the manner in which providers end the encounter, ensuring patients understand treatment plans, fosters more amicable physician-patient relationships [38, 39]. Given that patients often seek medical attention due to discomfort and may lack medical expertise, creating a clear understanding at the conclusion of the encounter is vital. This approach minimizes information asymmetry and positively influences the quality of diagnosis [40]. Interestingly, our study suggests that the dimension of setting the stage has a limited impact on healthcare quality in rural Chinese primary care settings. This may be attributed to the prevailing paternalistic doctor-patient relationship in rural China, where patients tend to be more reliant on doctors and may not expect them to fully understand and care about their personal concerns. This distinctive feature in patient expectations may explain the variation in our findings compared to those typically observed in Western contexts [40, 41].

### The heterogeneity analysis by physicians’ clinical knowledge level and training

The heterogeneity analysis conducted in our study unveiled significant variations in the impact of provider-patient communication on healthcare quality and patient satisfaction, contingent upon physicians’ clinical knowledge level and training. First, physicians with lower clinical knowledge levels may compensate for their knowledge deficit through stronger communication skills. In situations where medical expertise is lacking, these physicians tend to exercise greater caution in avoiding specialized terminology, preferring caring, empathetic, and supportive language that effectively conveys cases [42]. This emphasis on clear communication facilitates the acquisition of detailed patient history and symptom information, contributing to the improved execution of necessary questions and exams [4, 17]. The increase in clinical knowledge, on the other hand, diminishes the positive influence on checklist completion but heightens the impact on accurate diagnoses. This dual effect is attributed to the dominant position and strong confidence exhibited by physicians with higher knowledge levels [17, 18]. Such physicians often prioritize delivering accurate diagnoses over the explanatory aspect of communication, emphasizing the need for a balanced approach [16, 17]. This observation aligns with findings from studies in developing countries, highlighting the significance of short consultation times and limited communication in influencing healthcare quality and patient satisfaction in primary healthcare centers [13]. In contrast, physicians with higher clinical knowledge levels, equipped with concise and effective medical inquiries, can effectively gather patient information, thereby reducing information asymmetry between physicians and patients. Consequently, they can effectively leverage the positive impact of eliciting information communication skills on healthcare services.

Secondly, among physicians who underwent more frequent training, the dimension of eliciting information within doctor-patient communication skills had a more pronounced positive impact on healthcare quality. Additionally, the dimension of setting the stage and understanding the patient’s perspective had a more pronounced positive impact on patient satisfaction. Within the realm of eliciting information, physicians play a pivotal role in guiding patients to articulate their health concerns and systematically comprehend elements such as medical history, current conditions, and factors influencing their illnesses [17, 31]. Actively engaging in training programs that encompass disease knowledge and doctor-patient communication skills empowers primary physicians to strengthen their grasp of the framework for collecting disease-related information. This results in more targeted inquiry content, a broader scope of questioning, and proficiency in employing appropriate questioning techniques [43]. Additionally, participation in training programs among primary physicians is vital for improving triage skills and patient understanding. The preparatory phase, crucial in the diagnostic process, was identified in interviews with primary physicians as a key stage where eagerness to treat or lack of focus significantly contributed to clinical errors during patient admissions [44]. Moreover, during the preparatory phase, physicians create a welcoming environment by greeting patients appropriately and engaging in broader conversations, fostering cooperation, and providing valuable background information for the doctor’s subsequent assessment and examination. Ultimately, providers who undergo multiple training sessions not only excel in reception etiquette but also adopt a more patient-centric approach, thereby surpassing those without frequent training. This advancement significantly enhances the quality of healthcare services and boosts patient satisfaction [43].

### Limitations

Our study has several limitations. Firstly, our study relied on audio recordings to assess provider-patient communication skills. The exclusion of nonverbal behaviors may have led to an underestimation of the true level of communication skills. Secondly, the use of standardized patients, while effective, is constrained by the portrayal of cases with fewer obvious physical symptoms and low-risk invasive examinations. This limitation may impact the generalization of our findings to scenarios with more complex presentations. Thirdly, this is a cross-sectional study and our results represent correlations rather than attributions of causality.

## Conclusion

To conclude, our study has illuminated a low level of communication skills, subpar healthcare quality, and patient satisfaction among primary providers in rural China. Importantly, our findings highlight the crucial significance of targeted communication skills, particularly in specific dimensions, in shaping healthcare quality and patient satisfaction in this context. This emphasizes the urgent requirement for strategic policy initiatives by the Chinese government, specifically tailored to incentivize primary physicians in rural regions. These policies should prioritize the systematic enhancement of provider-patient communication skills as a key strategy for advancing healthcare quality and elevating patient satisfaction.

## Data Availability

Public availability of data would compromise privacy of participants. Data will be made available from the corresponding author on reasonable request.

## Footnotes

1 In practical applications, the logistic model may exhibit greater robustness, particularly when dealing with small sample sizes or rare events, thus enhancing the reliability of its estimates (see Greene W, 2008).
